# Attitude and Perceived Barriers Among Highly Educated Women Towards Cervical Cancer Screening by Pap Smear: An Online Survey

**DOI:** 10.7759/cureus.28641

**Published:** 2022-08-31

**Authors:** Mukta Agarwal, Sudwita Sinha, Geetika Singh, Shruti Singh, Shamshad Ahmad

**Affiliations:** 1 Department of Obstetrics and Gynaecology, All India Institute of Medical Sciences, Patna, Patna, IND; 2 Department of Family and Community Medicine, Netaji Subhas Medical College and Hospital (NSMCH) Bihta, Patna, IND; 3 Department of Family and Community Medicine, All India Institute of Medical Sciences, Patna, Patna, IND

**Keywords:** barriers, determinants, practice, perception, cancer cervix

## Abstract

Background

Cervical cancer continues to pose a heavy burden on developing countries like India. Early detection of precancerous lesions via Pap smear screening can greatly avert cervical cancer deaths. However, the uptake of cervical cancer screening is poor, and several barriers exist to adequately utilizing screening services. Knowledge of women’s attitudes in the target community is essential for successfully implementing a cervical cancer screening program.

Aim

This study aimed to provide insight into the attitude and perceived barriers among highly educated women and determine the association between the sociodemographic characteristics and their attitude towards screening.

Methods

It was an online descriptive study using a questionnaire conducted among highly educated women. Sociodemographic details and the perceived gynecological morbidities were enquired upon. The attitude was measured on a 5-point Likert scale, while practice was assessed by response towards ever screened. Significant barriers to not undergoing cervical cancer screening and determinants of attitude towards screening were evaluated.

Results

A total of 150 women participated, with a mean age of 36.9+9.7 years. Most (85.33%) women were apparently asymptomatic. Overall, the majority (82.67%) of participants had a favorable attitude toward cervical cancer screening, but only 5.33% of women were ever screened in the past. A major impediment to adequate practice identified was that a Pap test is ‘not required.’ In addition, the women’s age, marital status, and education were found to be significantly associated with women’s attitudes towards screening.

Conclusion

The study revealed that educated women do possess a favorable attitude towards cervical cancer screening. However, a major gap is still a hindrance between women’s perception and practice. This reiterates the need for a well-designed health educational program focusing on effective information, education, and communication (IEC) strategies and strengthening the national screening program by effectively incorporating it into the health system.

## Introduction

Globally, cervical cancer is the leading cancer among women, with an estimated 570,000 new cases and about 311,000 deaths in 2018 [1]. Though cervical cancer is seen to decrease in developed countries, developing countries are still burdened by this disease. For example, India contributes to 20% of the world’s cases [2], of which more than three-fourths are first seen at advanced stages with a poor probability of a good outcome [3]. Fortunately, cervix cancer is a preventable disease with a pre-malignant stage that usually occurs in younger women under the age of 40. A total of 80% of cervical cancers can be prevented by screening resulting in early diagnosis and treatment [4]. Screening techniques include liquid-based cytology with co-testing for human papillomaviruses (HPV) and visual inspection of the cervix with acetic acid and Lugol’s iodine. A highly sensitive and specific Pap smear is the most widely practiced method to detect pre-malignant lesions of the cervix. However, the percentage of women being screened by Pap smear is only 5% in India as only those women visiting gynecologists due to any reason are screened. Hence, asymptomatic women are never screened in their life [5,6]. Further, several barriers exist which hamper the rate of cervical screening, such as inaccessible facilities, false belief that the test is unnecessary or risk being low, fear of being diagnosed, embarrassment, ignorance, low socioeconomic status, lack of female health care providers, and busy schedules [7]. Recently, numerous studies have been reported from various parts of India on awareness of cervical cancer screening and its determinants among women. However, the majority of these studies have explored mainly the knowledge component of cervical cancer. They have focused either on the “nursing staff” working in hospitals where good awareness was seen though only 7%-8% of women had ever done a Pap test [8,9,10] or on the less literate women in the rural community where awareness of about 72% was seen. However, only 2%-6.9% ever had a Pap smear [11,12]. This clearly reflects that despite having adequate knowledge about cervical screening or being highly educated enough to comprehend the significance of cervical screening, women lack a positive attitude towards screening resulting in poor outcomes of screening. Knowledge of women’s attitudes in the target community is essential for successfully implementing a cervical cancer screening program [12]. Evaluating factors related to women’s attitudes will not only help remove the barriers to screening but would also be essential in developing specific strategies to increase its coverage. Hence, this study attempts to provide insight into the attitude and perceived barriers among highly educated women who can understand the significance of timely cervical screening and determine the association between the socio-demographic characteristics and their attitude towards cervical cancer screening by Pap smear.

## Materials and methods

It was an online questionnaire-based study conducted among highly educated women residing in different states of India. The study period was from January to February 2020. Using Google forms, a link was generated and circulated using Whatsapp to all the eligible women identified by the investigators. In addition, written informed consent to participate in the study was sought from all the eligible women.

Eligibility criteria

The women included in the study were between 21 and 60 years of age, sexually active, and educated up to at least graduation (either degree or diploma) or above. They gave consent to participate in the study.

The survey questionnaire consisted of the following study instruments: (1) Sociodemographic details like age, education, occupation, and area of residence; (2) Questions regarding any complaints related to the reproductive tract of women and the time period of being sexually active; (3) Attitude was measured on a 5-point Likert’s scale. Eight statements about cervical cancer screening having responses ranging from strongly agree to strongly disagree were given. Participants showing a positive attitude to any four statements were considered to have a favorable attitude; (4) Response to having ever undergone cervical screening was used to assess practice; (5) Participants with no history of prior screening were asked to mention the most important barriers. 

Statistical analysis

SPSS version 22.0 was used for statistical analysis of data. Mean, SD, frequency, and proportion were used as appropriate. Determinants of attitude were determined using the Chi-square test. P-value <0.05 was considered significant.

Ethical considerations

Since the study was only an online form-based survey, it was granted exemption from the requirement of ethics approval.

## Results

A total of 150 women participated, with a mean age of 36.9+9.7 years. A maximum of 132 (88.0%) women were Hindus and were educated up to postgraduate level or beyond (70.67%). Most participants were married (76.0%), and around three-fourths of 112 (74.67%) were employed (Table 1).

**Table 1:** Sociodemographic profile of participants (N=150).

Characteristics	Number (%)
Age (years) (mean+SD)	36.9+9.73
Religion	
Hindu	132 (88.0)
Muslim	4 (2.67)
Christian	6 (4.0)
Others	8 (5.33)
Education	
Graduate	44 (29.33)
Postgraduate and above	106 (70.67)
Occupation	
Unemployed	38 (25.33)
Employed	112 (74.67)
Marital Status	
Unmarried	34 (22.67)
Married	114 (76.0)
Divorced/Separated	2 (1.33)

Among the various gynecological morbidities as perceived by the participants, the majority, i.e., 128 (85.33%) women, had no complaint related to the reproductive tract. However, 12 (8%) women complained of heavy bleeding at menstruation, followed by vaginal discharge in eight (5.33%) cases. Table 2 describes the attitude of respondents towards cervical cancer screening according to the Likert scale. The majority of women agreed that ‘Any adult woman including you can get cancer cervix’, ‘All women including you should get a screening (Pap test) of cancer cervix’, ‘Pap test can detect changes in the cervix before it becomes cancer’, and ‘Pap test causes no harm to the women’. Around half (48%) agreed to ‘I may go for a pap test this year’. However, a quarter of women (26.66%) felt that ‘Getting a pap test done is embarrassing!’ while 20% believed that ‘Pap test is expensive’.

**Table 2:** Attitude of study participants regarding cervical cancer and its screening (N=150).

Attitude Variables	Level of Agreement (Likert scale)				
	Strongly agree No. (%)	Agree No. (%)	Neither agree nor disagree No. (%)	Disagree No. (%)	Strongly disagree No. (%)
Any adult woman including you can get cervix cancer	66 (44)	68 (45.33)	12 (8.0)	4 (2.67)	0 (0)
All women including you should get a screening (Pap test) of cervix cancer	88 (58.67)	54 (36.0)	8 (5.33)	0 (0)	0 (0)
Pap test can detect changes in the cervix before it becomes cancer	80 (53.33)	62 (41.33)	8 (5.33)	0 (0)	0 (0)
Pap test causes no harm to the women	78 (52.0)	52 (34.67)	20 (13.33)	0 (0)	0 (0)
I may go for a pap test this year	28 (18.67)	44 (29.33)	66 (44.0)	10 (6.67)	2 (1.33)
I may prefer for a screening test if it can be done with menstrual blood sample	24 (16.0)	44 (29.33)	52 (34.67)	18 (12.0)	12 (8.0)
Getting a pap test done is embarrassing!	8 (5.33)	32 (21.33)	46 (30.67)	46 (30.67)	18 (12.0)
Pap test is expensive	0 (0)	30 (20.0)	58 (38.67)	28 (18.67)	34 (22.67)

Based on the criteria of positive attitude, the majority had a favorable attitude toward cervical screening (124, 82.67%). Regarding actual practice, only eight (5.33%) women were ever screened for cancer cervix (Table 3).

**Table 3:** Adequacy of attitude and practice regarding cervical cancer and its screening (N=150).

Variable	Number (%)
Attitude	
Favorable	124 (82.67)
Not favorable	26 (17.33)
Practices	
Ever screened	8 (5.33)
Never screened	142 (94.67)

Table 4 shows the sociodemographic determinants of attitude among women. Women in the higher age group (46-60 years) had a significantly favorable attitude (91.67%) compared with those women belonging to 30-45 years of age (86.96%) and those below 30 years (64.71%) (p = 0.006). Regarding educational status, the respondents who were postgraduates or above had a more favorable attitude (88.68%) in comparison to graduates (68.18%), which was significant (p=0.005). Similarly, married women had a much more positive attitude (87.72%) than unmarried (70.59%) and divorced women, and this relation was also found to be statistically highly significant (p=0.0005). However, no significant difference was observed with respect to their religion and occupation.

**Table 4:** Association between sociodemographic factors with the attitude of the respondents. *: Statistically significant.

Characteristics	Attitude		P-value
	Favorable (N/%)	Not favorable (N/%)	
Age (years)			0.006*
<30	22 (64.71)	12 (35.29)	
30-45	80 (86.96)	12 (13.04)	
46-60	22 (91.67)	2 (8.33)	
Religion			0.11
Hindu	108 (81.82)	24 (18.18)	
Muslim	2 (50.0)	2 (50.0)	
Christian	6 (100.0)	0 (0.0)	
Others	8 (100.0)	0 (0)	
Education			0.005*
Graduate	30 (68.18)	14 (31.82)	
Postgraduate and above	94 (88.68)	12 (11.32)	
Occupation			0.15
Unemployed	28 (73.68)	10 (26.32)	
Employed	96 (85.71)	16 (14.29)	
Marital status			0.0005*
Unmarried	24 (70.59)	10 (29.41)	
Married	100 (87.72)	14 (12.28)	
Divorced/separated	0 (0)	2 (100.0)	

The most common barrier perceived by the women was that cervical cancer screening was ‘Not required’ (62, 41.33%), followed by ‘embarrassment’ in 26 (17.33%) cases. The other reasons cited for not undergoing screening were ‘lack of time’ (20, 13.33%), ‘lack of facilities for screening’ (14, 9.33%), ‘fear of pain’ (12, 8.00%), ‘social stigma’ (10, 6.67%) and other reasons (6, 4%) like ‘disapproval from family/husband.’

## Discussion

The present study explored the attitude regarding cervical cancer screening and the barriers perceived by educated women through an online-based survey. The sociodemographic findings correspond with the results obtained from a study conducted in New Delhi, where a majority of the women (86.7%) belonged to the Hindu religion, 79.3% were married, and the mean age of respondents was 35.87+ 12.11 years [13]. Cervical cancer occurs in women between the ages of 40 and 50 years, and its precursor lesion is 5-10 years earlier; hence, this age group is most suitable for screening [14]. Every woman should get a Pap test done before the age of 45 years at least once in her life [14,15]. Regarding complaints related to the reproductive tract, a majority (85.33%) of women in the current study had apparently no symptoms, which is in contrast to the studies by Mishra P et al. and Shaki O et al., where only 16% and 25.6% of women were found to be asymptomatic, respectively [16,17]. The most common gynecological morbidity in these studies was vaginal discharge (52% and 41.5%, respectively), contrary to our study (5.33%). The reason could be the higher educational status of our study participants resulting in better genital hygiene and proper sanitation. According to the present study, 89.33% of respondents agreed that they might be susceptible to cervical cancer, as against 29.2% in the study by Narayana G et al. [18]. Furthermore, none of the women in our study disagreed with the ‘Pap test causes no harm to the women’, which is different from this study [18], where around half of the women (49.6%) thought that screening causes harm to the client. Likewise, only 20% of women in the present study believed that ‘Pap smear is expensive’ in contrast to 51.9% in the above study [18]. The sharp differences in attitudes may be attributed to higher literacy and employment levels in our study population.

Overall, most women (82.67%) showed a favorable attitude towards cervical cancer screening, comparable to the study by AIIMS, Bhopal [19], where 80.5% of women had a positive attitude. Another study among community healthcare workers in the Varanasi district also reported an adequate attitude (93.9%) [20]. However, coming to the screening practices, only 5.33% of women in our study were ever screened by Pap smear in their lifetime, which coincides with the screening prevalence estimated for developing countries by the WHO (5%) [5]. Furthermore, inadequate practices have also been demonstrated by the above two studies, where only 9.5% and 8.3% of women were screened, respectively [19,20]. A major impediment to adequate practices of cervical cancer screening identified among these women was that a Pap test was ‘not required’ unless prescribed by the doctor or being symptomatic, which is congruent to several other studies [9,18,20,21]. This suggests that even well-educated women do not understand the rationale for screening. Moreover, the development of symptoms in cervical cancer is late, and the benefits of screening remain unachievable if they wait for symptom development. Hence, there is a pressing need to spread awareness regarding Pap smear screening through the dissemination of authentic information via mass media and awareness campaigns that will definitely assist in improvising the health-seeking behavior of women for cervical cancer screening.

Since attitude towards cervical cancer screening tests has a marked impact on its real practice, evaluating the determinants of women’s attitudes is of paramount importance. In our study, the various sociodemographic factors, such as age, marital status, and education of the women, were found to be significantly associated with their attitude towards screening (p<0.01). As the age advanced, the women developed a more favorable attitude towards screening. This finding is similar to other studies [19,21]. This may be because as age progresses, women are more likely to approach health care facilities for different types of reproductive morbidities when opportunistic screening is also done. Further, our finding that married women are more likely to have a positive attitude towards screening is similar to other studies in India [19,22]. Also, those women with higher educational status have a much more positive attitude toward cervical cancer and are more likely to avail the screening services. This association has been found to be highly significant and is in concordance with numerous studies [12,18,19,21]. Owing to better education and working status, women develop superior communication skills, more access to knowledge about screening tests through social interaction, and improved ability to absorb useful information leading to enhanced perception of the concept and benefits of screening by Pap smear. This reiterates the need for a well-designed health educational program focusing on effective Information, Education & Communication (IEC) strategies, including a multipronged approach and personal communication on cervical cancer screening in order to yield lasting results.

Limitations

The main limitation of this study is its small sample size and one-sided opinion without proper interaction. Also, cervical cancer is a disease of the lower socioeconomic status, but since this study involved an online-based questionnaire, it is obvious that participants would be mostly of higher socioeconomic status with access to new technologies and the ability to operate smart technologies and not the lower socioeconomic status women. Also, participants may seek help from someone else while answering the questionnaire. So the study population may not be actually representative of the disease population.

## Conclusions

The study revealed that educated women possess a favorable attitude (strong agreement on all the appropriate attitude variables regarding cervical cancer and its screening) towards cervical cancer and its prevention but do not transform it into actual practice. The mere availability of Pap smear is not sufficient for the control of cervical cancer; it should be effectively incorporated into the health system along with strengthening the national screening program. Addressing the various barriers and minimizing missed opportunities for Pap smear screening is essential. Thus, the awareness and adequate utilization of screening services by the target population shall ultimately determine the impact of health promotion in decreasing morbidity and mortality of cervical cancer. Further studies on the utilization rate of screening services, where available, can provide insight into whether mass screening of cervical cancer can be properly implemented in the target population.
